# Occurrence Dynamics and Chemical Control of *Mycterothrips glycines* in Soybean Field in Northeast China

**DOI:** 10.3390/insects17040365

**Published:** 2026-03-28

**Authors:** Yue Zhou, Tianhao Pei, Xiaoshuang Li, Liyan Zhang, Zhengxiao Du, Yijin Zhao, Long Wang, Yu Gao

**Affiliations:** 1 College of Plant Protection, Jilin Agricultural University, Changchun 130118, China; 2 Key Laboratory of Soybean Disease and Pest Control, Ministry of Agriculture and Rural Affairs, Changchun 130118, China

**Keywords:** chemical control, insecticide, soybean pest, thrips, toxicity

## Abstract

*Mycterothrips glycines* Okamoto (1911) (Thysanoptera: Thripidae) is rapidly increasing in fields, posing a new and potentially serious threat to soybean production in Northeast China. To clarify the population dynamics and screen effective insecticides against *M. glycines*, systematic monitoring and pesticide evaluation were conducted from 2024 to 2025. Occurrence dynamics were continuously monitored using yellow and blue sticky boards, while ten commonly used commercial insecticide formulations (thiamethoxam, clothianidin, sulfoxaflor, acetamiprid, imidacloprid, fenthion, pyridaben, abamectin, beta-cypermethrin, spinetoram) were hierarchically screened through laboratory bioassays, pot trials, and field spraying experiments. The results revealed a distinct ‘rise-and-fall’ occurrence pattern, with the initial, peak, and late occurrence periods occurring in mid-July, late July to early August, and mid-to-late August, respectively. Thiamethoxam and clothianidin exhibited the strongest toxicity against *M. glycines* (LC_50_ values of 12.87 mg/L and 13.46 mg/L, respectively), achieving field control efficacies exceeding 85%, which were significantly superior to conventional agents such as imidacloprid and abamectin. The study identified the soybean flowering stage as the critical window for control, recommending preventive interventions when sticky trap monitoring indicates the initial population peak (around mid-July). This research fills the technical gaps regarding the occurrence dynamics of *M. glycines* and the lack of registered control products in China, providing essential support for precision monitoring and management of soybean thrips.

## Simple Summary

*Mycterothrips glycines* (Thysanoptera, Thripidae) recently emerged as a new and potential pest in soybean fields in Northeast China. This study investigated the population dynamics of *M. glycines* and evaluated ten commonly used commercial insecticide formulations (thiamethoxam, clothianidin, sulfoxaflor, acetamiprid, imidacloprid, fenthion, pyridaben, abamectin, beta-cypermethrin, spinetoram) through laboratory bioassays, pot trials, and field experiments. The results showed a distinct ‘rise-and-fall’ occurrence pattern, with peak populations occurring from late July to early August. Thiamethoxam and clothianidin exhibited the highest toxicity and field control efficacy, exceeding 85%, significantly outperforming conventional agents such as imidacloprid and abamectin. The study identified mid-July as the critical timing for preventive control based on sticky trap monitoring. These findings provide essential technical support for integrated management of soybean thrips.

## 1. Introduction

**Figure 1:**
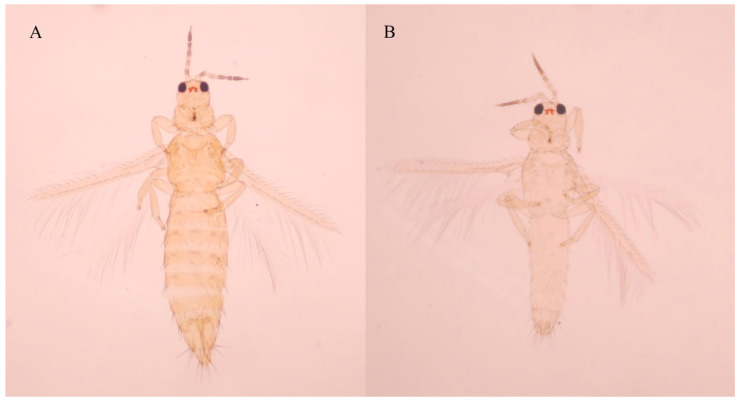
Slide specimens of *Mycterothrips glycines*: (**A**) Female adult; (**B**) male adult. Photo by Tianhao Pei.

Thrips in soybeans are not often noticed. Owing to their small size and cryptic feeding behavior, herbivorous thrips were historically overlooked and considered minor pests in soybean fields [1,2,3]. In recent years, however, thrips have emerged as a key constraint to the high-quality and high-yield production of soybeans, with outbreaks becoming more frequent and economically damaging across major soybean-growing regions. In North America, *Neohydatothrips variabilis* (Beach) (Thysanoptera: Thripidae), *Frankliniella tritici* (Fitch) (Thysanoptera: Thripidae) and *F. fusca* (Hinds) (Thysanoptera: Thripidae) are the dominant species, transmitting soybean vein necrosis virus that markedly reduces seed quality and soybean yield [4,5,6]. In Brazil, *F. schultzei* and *Caliothrips phaseoli* (Hood) (Thysanoptera: Thripidae) cause yield losses exceeding 15% in key production areas [2,3,7]. In Northeast China, *Thrips nigropilosus* Uzel (Thysanoptera: Thripidae), *Thrips flavus* Schrank (Thysanoptera: Thripidae) and *Thrips tabaci* Lindeman (Thysanoptera: Thripidae) are now recognized as significant pests [8,9,10]. Recent surveys reveal a further threat: the thrips *Mycterothrips glycines* Okamoto (1911) (Thysanoptera: Thripidae), previously scarcely recorded, is increasing rapidly in soybean fields (Figure 1). *M. glycines* is a relatively new soybean threat in Northeast China. White to yellow spots may appear on soybean seedling leaves as a result of thrips feeding. This damage can lead to poor plant vigor and stunted growth. Seedlings may die under heavy infestations of soybean thrips, which can also cause leaf crinkling. There is currently no record indicating that *M. glycine* itself is a vector for the transmission of plant viruses. This pest has been detected in most of the soybean-producing areas in Northeast China. The occurrence of *M. glycines* in soybean thrips could be closely related to the cultivation methods, such as soybean area expansion, continuous cropping, or compact planting. Under suitable conditions (e.g., hot and dry), *M. glycines* may develop locally and cause significant damage to soybean leaves or yield. *M. glycines* has a wide range of host plants, posing a significant potential threat. This pest not only feeds on legume crops (e.g., *Glycine max* (L.) Merr. (Fabales, Fabaceae), *Phaseolus angularis* Linn. (Fabales, Fabaceae), *Vigna unguiculata* (L.) Walp. (Fabales, Fabaceae)) in China [11,12,13], but also on fruit trees (*Malus pumila* (Suckow) Borkh.) in Andong region, South Korea [14] and vegetable crops (e.g., *Solanum melongena* L. (Solanales, Solanaceae), *Cucumis sativus* L. (Cucurbitales, Cucurbitaceae), *Vitis vinifera* L. (Vitales, Vitaceae), *Morus australis* Poir. (Rosales, Moraceae)) in Okayama, Japan [15,16,17]. Previous studies have shown that during the flowering stage of soybeans, the population of *M. glycines* is relatively high and often occurs in combination with thrips such as *Frankliniella intonsa* (Trybom) (Thysanoptera: Thripidae) in Changchun [12,13]. However, the population dynamics and key occurrence periods of this pest throughout its entire soybean growth period are still unclear. Currently, there is no insecticide product specifically registered for controlling pest thrips infesting soybean crops in China [9], leaving soybean growers without legal or validated management options. Urgent priorities are therefore (i) to quantify the field population dynamics of *M. glycines* across the soybean growth cycle, and (ii) to generate efficacy data for commonly available insecticides, thereby supporting both scientific advice and future product registration.

## 2. Materials and Methods

### 2.1. Insect

*Mycterothrips glycines* adults were collected from the soybean field of Jilin Agricultural University (43°48′7″ N, 125°24′51″ E, Changchun, Jilin Province). The field plot was not sprayed with chemical insecticides. Adult thrips were captured using the sweeping net. The specimens were then identified as *M. glycines* under a stereomicroscope in the laboratory [11]. The thrips were then fed soybean leaves (cv. ‘Jiyu303’) in a constant temperature incubator at 25 ± 1 °C, with 70 ± 10% relative humidity (RH) and a 16 h:8 h photoperiod for more than one generation [10]. To confirm the identity of females, individual thrips were examined under a stereomicroscope to observe the distinct ovipositor at the end of the abdomen [15].

### 2.2. Methods for Investigating the Occurrence Dynamics in Soybean Field

The experiment was conducted from June to September 2024 and from May to October 2025. The planting density was 250,000 plants per hectare. The occurrence dynamics of *M. glycines* was investigated by colored sticky boards in a soybean field (cv. ‘Jiyu303’). The yellow and blue sticky boards (25 cm × 20 cm) were purchased from Zhangzhou Ingle Agricultural Technology Co., Ltd. (Zhangzhou, China). After the seedlings emerged, blue and yellow colored sticky traps were hung in the experimental area, with six replicates for each color. The straight-line distance between traps of different colors exceeded 15 m, and sticky boards of the same color were spaced more than 10 m apart to ensure no interference among traps. Yellow sticky boards were suspended 10–15 cm above the soybean canopy. After 24 h, the sticky boards were removed and taken to the laboratory for thrips counting. Colored sticky boards were re-hung every other day; surveys were postponed or rescheduled in case of rain or hail.

### 2.3. Insecticides

Ten commercial insecticide formulations were selected based on farmers’ usage habits. All insecticides tested were registered for use on various crops in China. Basic information on the chemical insecticides is thiamethoxam, clothianidin, sulfoxaflor, acetamiprid, imidacloprid, fenthion, pyridaben, abamectin, beta-cypermethrin, spinetoram, and shown in Table S1.

### 2.4. Methods of Laboratory Bioassay

The toxicity bioassay for ten insecticides against *M. glycines* followed the protocol outlined by Pei et al. [9]. Fresh foliage from soybean cultivar ‘Jiyu303’ was excised into approximately 5.0 cm × 2.5 cm, washed with distilled water, and left to dry under ambient air for 15–20 min. Serial dilutions of each insecticide were prepared to five different concentrations (Table 1).

The soybean leaf segments were dipped in these preparations for 10 s, then allowed to dry under ambient conditions. Subsequently, thirty 2-day-old female adults of *M. glycines* were transferred into 50 mL plastic centrifuge tubes through the centrifuge port. Apertures were sealed with Parafilm membrane perforated with ~80 pinholes to facilitate gas exchange (Figure S1). The treated thrips were then kept under controlled conditions (25 ± 1 °C, 70 ± 5% relative humidity, 16 h: 8 h light: dark cycle). Each treatment level, including the untreated check, was replicated three times. The number of dead thrips was recorded after 24 h. Equations (1)–(3) were used for calculating the mortality rate, control-corrected mortality, and relative bioactivity index, respectively [9].

(1) $$R_{1}=\frac{n_{1}}{N}\times 100$$

(2) $$R_{2}=\frac{T_{2}-T_{1}}{1-T_{1}}\times 100$$

(3) $$R=\frac{V_{2}}{V_{1}}\times 100$$

Here, *R*_1_ denotes the mortality proportion; *n*_1_ is the number of dead thrips; *N* denotes the total treated thrips number; *R*_2_ denotes the adjusted mortality; *T*_1_ represents the spontaneous death rate in untreated controls; *T*_2_ denotes the treatment mortality rate; *R* denotes the relative bioactivity index; *V*_1_ denotes the LC_50_ value of the test insecticide; *V*_2_ denotes the LC_50_ value of the standard insecticide, using the insecticide with the highest LC_50_ value among all insecticides as the reference compound, and its relative bioactivity index is defined as 1.

### 2.5. Methods of Pot Trials

The insecticide efficacy of ten insecticides against *M. glycines* was evaluated following the method described by Pei et al. [9]. In the pot trial, soybean (cv. ‘Jiyu303’) was grown in 13.5 cm wide × 10 cm high plastic pots, each containing ≈700 g soil plus 4 g sulfur-coated slow-release fertilizer (Liaoning Anshan Sinuo Chemical Co., Ltd., Anshan, China). Nine seeds were sown per pot and kept in a greenhouse until the second pair of true leaves appeared (~15 days). After thinning to one plant per pot, a cardboard collar was fitted around each stem to stop dead thrips from falling onto the substrate; no further watering was given. Using laboratory bioassay data, five insecticide solutions were prepared in distilled water at the predetermined concentrations (Table 2).

Dilutions were calibrated to match a field dose of 900 L water ha^−1^ of soybean. Per concentration, 100 mL of solution was made; 5 mL was sprayed onto each pot with a hand sprayer, while controls received distilled water. After the film dried, each pot was capped with mesh gauze, and 30 female adults (2-day-old) were released. Thrips were aspirated from the culture cage into centrifuge tubes and then emptied onto the plants. Every concentration, including the control, was tested on 30 females with three replicates. Pots were set 1 m apart in a randomized block design under greenhouse conditions. Mortality was scored 1, 3 and 7 days after treatment, and efficacy was computed with Equation (4) [18].

(4) $$E=\left(1-\frac{N_{2}\times C_{1}}{C_{2}\times N_{1}}\right)\times 100$$

where *E* is the insecticide efficacy, *N*_1_ and *N*_2_ are the thrips number in the treated group before and after application, and *C*_1_ and *C*_2_ are the corresponding numbers in the control group.

### 2.6. Field Spraying Experiment

A field spraying experiment was conducted from 13 July 2025 to 27 July 2025, at the soybean experimental field (43°48′13′′ N, 125°22′57″ E). Plots measuring 20 m^2^ were established using the soybean variety ‘Jiyu303’. Plants were arranged in rows spaced 65 cm apart, with 15 cm between individual plants and 2 m between plots. The total sowing density was 14,670 plants/667 m^2^ (Figure S2). The plots were laid out in a randomized block design with standard agronomic practices except for the use of pesticides. Four products—imidacloprid, fenthion, sulfoxaflor and cyetpyrafen—chosen for high laboratory/pot activity were tested at five concentrations each, as follows: thiamethoxam 1.62, 3.24, 4.86, 6.48, 8.10 g ai/ha; clothianidin 2.60, 5.18, 7.78, 10.37, 12.96 g ai/ha; imidacloprid 6.30, 12.60, 18.90, 25.20, 31.50 g ai/ha. All sprays were diluted in 900 L of water per 1 ha and applied with a hand sprayer (Delixi Group Co., Ltd., Shanghai, China) at 0.15–0.4 MPa via a wand fitted with whirlpool nozzles without filters; tap water served as the control. Efficacy was assessed by shaking 20 randomly selected plants per plot over a plastic disk and counting dislodged thrips 1 day before and 1, 3, 7, and 14 days after treatment. Each concentration and the control were replicated three times; population decline and field efficacy were calculated with Equations (5) and (6) [19].

(5) $$P=\frac{P_{0}-P_{1}}{P_{0}}\times 100$$

(6) $$E_{0}=\frac{PR_{1}-PR_{0}}{1-PR_{0}}\times 100$$

where *P* is the population decline rate; *P*_0_ and *P*_1_ denote pest numbers before and after application, *E*_0_ is the field efficacy, and *PR*_0_ and *PR*_1_ are the decline rates in the control and threat areas, respectively.

### 2.7. Data Analysis

Mortality data were analyzed by probit analysis to obtain the log-dose–response, slopes, 95% confidence intervals, and the median lethal concentration (LC_50_) in mg L^−1^. Control-corrected mortality was used. All calculations were carried out with DPS 20.05 software (Hangzhou Ruifeng Information Technology Co., Ltd., Hangzhou, China) [20]. Lower LC_50_ values indicate greater relative bioactivity and greater potency. The correlation coefficient was used to evaluate goodness-of-fit, with larger values reflecting a better fit. Model reliability was verified through the chi-square test; smaller chi-square values signified less deviation between observed and expected results. Statistical differences between two insecticides were considered significant when their 95% confidence intervals did not overlap.

A one-way ANOVA was run in IBM SPSS Statistics 26.0 (International Business Machines Corporation, Armonk, NY, USA), and Tukey’s post hoc test (*p* < 0.05) identified significant differences among treatment means. Logistic equation fitting analysis of the daily cumulative population of thirps was performed using Python 3.10.11 (Python Software Foundation, Wilmington, DE, USA). The NumPy and Pandas modules were used to conduct statistical calculations on the population dynamics data of *M. glycines* monitored by yellow/blue sticky boards during 2024–2025, followed by the elimination of outliers and verification of data distribution characteristics. Then, the scipy.optimize.curve_fit function from the SciPy library was adopted to fit the parameters of the logistic equation for the population data via the nonlinear least squares method, and the core parameters of the model were solved. Finally, the coefficient of determination (*R*^2^) was used as a quantitative indicator to evaluate the fitting results of the logistic equation for the population dynamics trend of *M. glycines*. The core ‘.polyfit’ function in the NumPy library was used to solve for the optimal polynomial coefficients that best fit the data using the least squares method, thereby deriving the fitted equation.

## 3. Results

### 3.1. Occurrence Dynamics in Soybean Field

**Figure 2:**
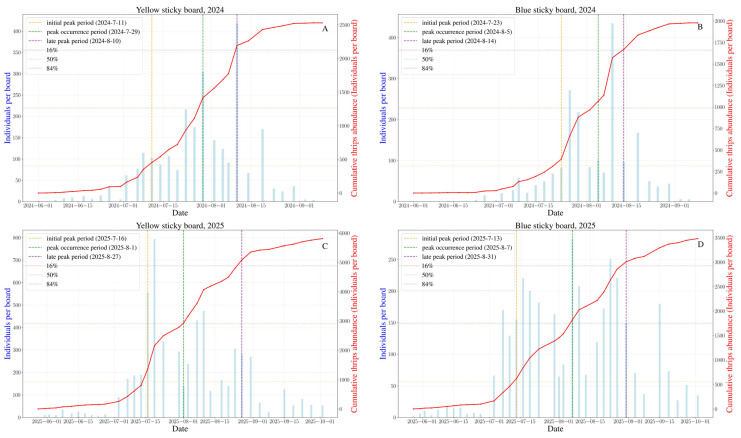
Occurrence dynamics of *Mycterothrips glycines* in soybean fields (Changchun, 2024 and 2025). (**A**) Yellow sticky board, 2024; (**B**) blue sticky board, 2024; (**C**) yellow sticky board, 2025; (**D**) blue sticky board, 2025.

The population dynamics of *M. glycines* were similar in 2024 and 2025, both showing a rise-and-fall pattern as the soybean season progressed (Figure 2). Numbers were low at the seedling stage in both years. After the flowering stage began, the thrips population increased, but the time sequence differed. In 2024, thrips increased slowly during flowering, then rose steadily once pods started to fill, peaking at 418.6 individuals per sticky trap on 10 August. In 2025, numbers surged rapidly during flowering and reached their maximum of 794.17 per trap on 19 July; although counts remained high during pod-fill, they were lower than the flowering peak. From September to October, as the crop matured, thrips declined and, despite lingering presence, stayed at low levels.

**Figure 3:**
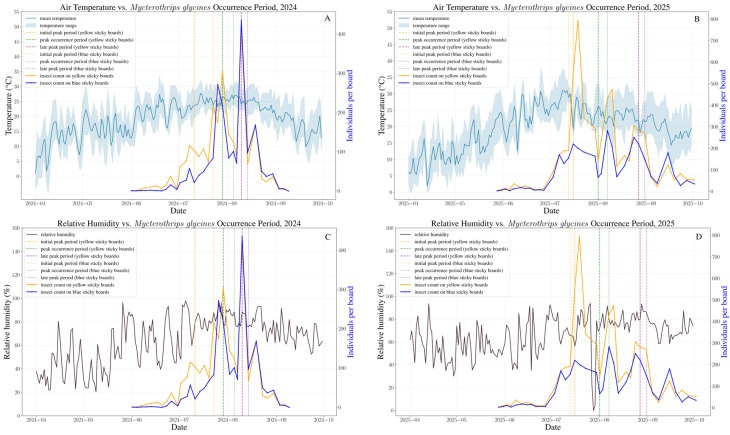
Occurrence dynamics of *Mycterothrips glycines* in soybean fields (Changchun, 2024 and 2025). (**A**) Air temperature vs. *M. glycines* occurrence period, 2024; (**B**) air temperature vs. *M. glycines* occurrence period, 2025; (**C**) relative humidity vs. *M. glycines* occurrence period, 2024; (**D**) relative humidity vs. *M. glycines* occurrence period, 2025.

The initial peak period, peak occurrence period, and late peak period were calculated and determined based on 16%, 50% and 84% of the total thrips population of *M. glycines*, respectively (Table 3). After fitting, the occurrence dynamics of *M. glycines* conform to the logistic equation (Figure S2). The mathematical expressions are shown in Table 3. The occurrence period remained largely consistent during 2024–2025. The initial peak occurred in mid-July. The peak occurrence period occurred from late July to early August. The late peak period occurred at mid-to-late August. Additionally, both colored sticky boards are effective at trapping *M. glycines*. The occurrence periods and numbers of thrips on the two coloured sticky boards differed, while the yellow sticky board is relatively better (Figure 3).

Regression analysis revealed that temperature and relative humidity each have a nonlinear relationship with population size, primarily taking the form of a quadratic function (Figure S3). In 2024, both temperature and relative humidity had a certain impact on thrips populations. However, in 2025, the nonlinear relationship between temperature and thrips population size was not evident, and the coefficients of determination obtained from the analyses for both years were generally low (Table S4). This suggests that while temperature and relative humidity influence thrips population size, other factors—such as the initial population density of the overwintering generation, the number of generations, or the presence of natural enemies—may play a more significant role.

### 3.2. Laboratory Bioassay

The toxicity of ten insecticides against *M. glycines* was shown in Table 4. Among the tested insecticides, thiamethoxam (LC_50_ value = 12.87 mg/L) exhibited the highest toxicity against *M. glycines*, followed by clothianidin (13.46 mg/L) and sulfoxaflor (15.72 mg/L). In contrast, spinetoram, beta-cypermethrin and abamectin showed relatively low toxicity, with LC_50_ values of 60.88, 50.73, and 29.36 mg/L.

### 3.3. Pot Trials

The insecticidal efficacy of different insecticides against *M. glycines* varied significantly over time and with increasing application concentrations in the pot trials (Table 5). Seven days after application, thiamethoxam, clothianidin, imidacloprid, and fenthion achieved more than 90% control efficacy against *M. glycines*. Comparing the control efficacy at 1, 3, and 7 days after application at the highest concentration revealed that the control efficacy of acetamiprid at 1.8 g ai/ha and beta-cypermethrin at 3.24 g ai/ha were significantly higher than imidacloprid at 31.5 g ai/ha, fenthion at 18.9 g ai/ha, and pyridaben at 10.8 g ai/ha (*F* = 7.281, *p* < 0.001). Three days after application, beta-cypermethrin at 3.24 g ai/ha was significantly higher than abamectin at 1.8 g ai/ha and fenthion at 18.9 g ai/ha (*F* = 3.026, *p* = 0.007). Seven days after application, there was no significant difference in the maximum concentration among the various pharmaceutical agents (*F* = 0.410, *p* = 0.932) (Table S3).

One day after application, the control efficacy of abamectin at 1.8 g ai/ha was (58.39 ± 4.16)%, significantly higher than that of 1.13 g ai/ha (40.15 ± 12.00)% and 0.90 g ai/ha (19.71 ± 9.30)% (*F* = 36.792, *p* < 0.001). The control efficacy of spinetoram at 4.86 g ai/ha was (66.42 ± 13.76)%, significantly higher than that of 3.24 g ai/ha (37.96 ± 9.30)% and 2.7 g ai/ha (27.74 ± 6.53)% (*F* = 7.428, *p* < 0.001). The control efficacy of acetamiprid at 10.13 g ai/ha was (75.18 ± 6.00)%, significantly higher than that of 5.63 g ai/ha (41.61 ± 9.30) % and 7.88 g ai/ha (54.01 ± 7.57)% (*F* = 60.901, *p* < 0.001). The highest control efficacy of sulfoxaflor was (67.88 ± 4.76)% at 5.94 g ai/ha, while the lowest was (13.87 ± 4.16)% at 1.98 g ai/ha (*F* = 72.252, *p* < 0.001). The efficacy of fenthion increased significantly with concentration, reaching (42.34 ± 7.02)% at 18.9 g ai/ha. The efficacy of pyridaben rose with increasing dose, with the highest value of (50.37 ± 8.40)% at 10.8 g ai/ha, significantly superior to the other rates (*F* = 41.854, *p* < 0.001). For imidacloprid, the control efficacy was (56.20 ± 9.30)% at 25.2 g ai/ha, followed by (50.37 ± 7.57)% at 31.5 g ai/ha, whereas 6.3 g ai/ha gave the lowest value of (29.93 ± 10.13)% (*F* = 3.046, *p* < 0.05). The best control provided by thiamethoxam was (64.23 ± 11.07)% at 8.1 g ai/ha, while the lowest was (31.39 ± 10.45)% at 1.62 g ai/ha, efficacy increasing with concentration (*F* = 7.427, *p* < 0.001). The highest efficacy of clothianidin was (64.23 ± 8.71)% at 12.96 g ai/ha, and the lowest was (25.55 ± 10.51)% at 2.60 g ai/ha, with efficacy rising as the dose increased (*F* = 11.519, *p* < 0.001). The control efficacy of beta-cypermethrin was greatest at 3.24 g ai/ha (70.80 ± 9.30)%, significantly higher than the other rates, and lowest at 1.22 g ai/ha (23.36 ± 7.74)% (*F* = 27.871, *p* < 0.001).

Three days after application, the highest control efficacy of abamectin was (58.39 ± 4.16)% at 1.80 g ai/ha, followed by (48.18 ± 8.05)% at 1.58 g ai/ha, while the lowest was (19.71 ± 9.30)% at 0.90 g ai/ha (*F* = 13.220, *p* < 0.001). Spinetoram gave the greatest control of (74.50 ± 8.13)% at 4.86 g ai/ha, significantly outperforming the other rates (*F* = 8.051, *p* < 0.001). Acetamiprid provided the best result of (84.25 ± 6.16)% at 10.13 g ai/ha, significantly higher than all other concentrations (*F* = 70.776, *p* < 0.001). Sulfoxaflor achieved the optimum control of (73.75 ± 4.28)% at 5.94 g ai/ha, followed by (67.75 ± 4.28)% at 4.95 g ai/ha, both significantly superior to the remaining doses (*F* = 62.524, *p* < 0.001). The efficacy of fenthion increased steadily with dose, differing highly significantly among rates; the best control was (70.75 ± 7.21)% at 18.90 g ai/ha (*F* = 45.827, *p* < 0.001). Pyridaben also showed a clear dose–response, with significant differences among gradients; the highest efficacy was (77.50 ± 5.93)% at 10.80 g ai/ha (*F* = 80.000, *p* < 0.001). Imidacloprid gave the greatest control of (78.25 ± 13.88)% at 25.20 g ai/ha, followed by (73.00 ± 8.13)% at 31.50 g ai/ha (*F* = 7.527, *p* < 0.001). Thiamethoxam performed best at 8.10 g ai/ha (78.25 ± 4.89)%, whereas the lowest efficacy was (46.75 ± 7.21)% at 1.62 g ai/ha (*F* = 12.308, *p* < 0.001). Clothianidin provided the highest control of (79.00 ± 8.22)% at 12.96 g ai/ha, significantly exceeding all other doses (*F* = 12.808, *p* < 0.001). Beta-cypermethrin achieved peak efficacy of (85.75 ± 5.56)% at 3.24 g ai/ha, while the lowest was (41.50 ± 5.03)% at 1.22 g ai/ha (*F* = 18.052, *p* < 0.001).

Seven days after application, abamectin again gave the best control of (89.74 ± 5.99)% at 1.80 g ai/ha, followed by (84.21 ± 8.83)% at 1.58 g ai/ha, whereas 0.90 g ai/ha yielded the lowest efficacy of (36.84 ± 10.06)% (*F* = 31.767, *p* < 0.001). Spinetoram reached its maximum of (87.37 ± 5.85)% at 4.86 g ai/ha, with the minimum of (44.74 ± 9.26)% recorded at 2.7 g ai/ha (*F* = 13.690, *p* < 0.001). Acetamiprid again showed the highest control of (86.58 ± 4.50)% at 10.13 g ai/ha, efficacy increasing consistently with dose (*F* = 16.677, *p* < 0.001). Sulfoxaflor achieved optimum control of (88.16 ± 8.83)% at 5.94 g ai/ha, followed by (79.47 ± 7.59)% at 4.95 g ai/ha, while 1.98 g ai/ha gave the lowest result of (37.63 ± 7.59)% (*F* = 38.660, *p* < 0.001). Fenthion provided its best efficacy of (90.53 ± 9.09)% at 18.90 g ai/ha, significantly higher than all other concentrations (*F* = 32.164, *p* < 0.001). Pyridaben again performed best at 10.80 g ai/ha (88.16 ± 6.24)%, followed by (78.68 ± 10.67)% at 9.45 g ai/ha (*F* = 38.828, *p* < 0.001). Imidacloprid gave the greatest control of (90.53 ± 4.50)% at 31.50 g ai/ha, whereas 6.30 g ai/ha produced the lowest efficacy of (51.84 ± 10.22)% (*F* = 7.940, *p* < 0.001). Thiamethoxam peaked at (91.32 ± 7.06)% when applied at 8.10 g ai/ha, while the lowest figure was (54.21 ± 10.29)% at 1.62 g ai/ha (*F* = 10.714, *p* < 0.001). Clothianidin again delivered the highest control of (92.11 ± 4.83)% at 12.96 g ai/ha, significantly surpassing all other doses (*F* = 12.404, *p* < 0.001). Beta-cypermethrin ME achieved its maximum efficacy of (89.74 ± 8.19)% at 3.24 g ai/ha, with the minimum of (55.79 ± 9.42)% at 1.22 g ai/ha (*F* = 9.500, *p* < 0.001).

### 3.4. Field Efficacy Experiment

The field efficacy of thiamethoxamand clothianidin against *M*. *glycines* shows a certain upward trend over time, but imidacloprid exhibited lower field performance (Table 6). The field efficacy of thiamethoxam at 8.10 g ai/ha (85.89 ± 2.91)% and clothianidin at 12.96 g ai/ha (80.94 ± 5.93)% was significantly higher than imidacloprid at 31.5 g ai/ha (42.16 ± 0.70)% (*F* = 12.027, *p* < 0.001).

One day after application, the control efficacy of thiamethoxam at 3.24 g ai/ha (66.13 ± 3.34)% was relatively better compared to other concentrations of this insecticide, followed by 4.86 g ai/ha (61.14 ± 5.94)% and 8.10 g ai/ha (59.05 ± 11.17)% (*F* = 8.595, *p* = 0.003); clothianidin at 12.96 g ai/ha showed the best control efficacy (69.20 ± 2.24)%, followed by 10.37 g ai/ha (62.58 ± 13.41)% (*F* = 3.842, *p* = 0.038); imidacloprid at 6.30 g ai/ha showed the best control efficacy (56.46 ± 5.87)%, followed by 18.90 g ai/ha (47.73 ± 7.84)% (*F* = 2.018, *p* = 0.168).

Three days after application, thiamethoxam at 8.10 g ai/ha showed the best control efficacy (67.73 ± 6.31)%, followed by 6.48 g ai/ha (64.78 ± 3.92)% (*F* = 1.889, *p* = 0.189); clothianidin at 7.78 g ai/ha showed the best control efficacy (44.75 ± 13.11)%, followed by 12.96 g ai/ha (44.12 ± 5.15)% (*F* = 1.093, *p* = 0.411); imidacloprid at 25.2 g ai/ha showed the best control efficacy (52.34 ± 4.57)%, followed by 31.5 g ai/ha (45.88 ± 5.56)% (*F* = 1.968, *p* = 0.176).

Seven days after application, thiamethoxam at 8.10 g ai/ha showed the best control efficacy (77.34 ± 5.50)%, followed by 3.24 g ai/ha (75.47 ± 0.32)% (*F* = 11.716, *p* < 0.001); clothianidin at 7.78 g ai/ha showed the best control efficacy (74.25 ± 1.80)%, followed by 12.96 g ai/ha (68.83 ± 6.73)%; imidacloprid at 31.5 g ai/ha showed the best control efficacy (51.22 ± 5.13)%, followed by 18.90 g ai/ha (45.50 ± 11.17)% (*F* = 1.143, *p* = 0.391).

Fourteen days after application, thiamethoxam at 8.10 g ai/ha showed the best control efficacy (85.89 ± 2.91)%, followed by 6.48 g ai/ha (63.11 ± 11.47)% (*F* = 7.130, *p* = 0.006); clothianidin at 12.96 g ai/ha showed the best control efficacy (80.94 ± 5.93)%, followed by 10.37 g ai/ha (79.52 ± 2.04)% (*F* = 2.736, *p* = 0.090); imidacloprid at 31.5 g ai/ha showed the best control efficacy (42.16 ± 0.70)%, followed by 25.2 g ai/ha (38.12 ± 1.89)% (*F* = 0.640, *p* = 0.646).

## 4. Discussion

Soybean thrips rarely cause severe production losses, but soybeans are most susceptible to thrips damage early in the growing stages. The objective of this study was to quantify the field population dynamics and identify effective insecticides against *M. glycines* and to lay the foundation for its integrated management. This study combines two consecutive years of systematic monitoring and regional historical data to reveal the new changes in the status of thrips pests in the spring soybean area of Northeast China. It is worth noting that Gao Yu et al. (2019) found *T. flavus* as the dominant species in the Changchun (accounting for 56.97% of the total population) in early investigations [12]. In this study, the population and harmful potential of *M. glycines* showed a rapid upward trend. Its mixed occurrence pattern with *F. intonsa* during the flowering stage (compared to the historical record of 8.04% proportion [12]) indicates that this species is transitioning from a companion species to a key pest species, which needs more attention. The observed pattern of ‘first rising and then falling’ in this study is basically consistent with the historical dynamic pattern of *T. flavus* [13], but *M. glycines* shows a more obvious dependence on the flowering stage. Previous reports have shown that the peak period of *T. flavus* is concentrated from the peak flowering stage to the pod-beginning stage [13], while this study further accurately located it from late July to early August through sticky board monitoring. This phenological synchrony may be related to the nutrient resources (pollen, nectar) provided by the tofu pudding apparatus and the protection of the microhabitat, which needs further research. The differences in occurrences between 2024 and 2025 may be caused by meteorological factors (Table S2). The high temperature and drought in summer are conducive to the occurrence of thrips, while the low temperature accompanied by rainfall can lead to a significant decline in population size [8,10,12]. More in-depth research will be conducted on the prediction and forecasting of thrips, providing a basis for guiding prevention and control.

The insecticides imidacloprid, thiamethoxam, clothianidin, acetamiprid, and sulfoxaflor used in this study all belong to the neonicotinoid group of insecticides [21]. Neonicotinoid insecticides are classified as the fourth generation of pesticides, following organophosphates, pyrethroids, and carbamates. Due to their broad-spectrum insecticidal activity, these compounds have been widely adopted globally [22]. The insecticidal activity of this class of insecticides against thrips has been extensively documented; they also demonstrated good efficacy in this study. Thiamethoxam applied at a concentration of 12 g/100 L water demonstrated effective control of *Scirtothrips dorsalis* Hood (Thysanoptera: Thripidae) infesting mango trees, achieving a mortality rate of 64.89% after 168 h of treatment [23]. This study found that the field spray efficacy of the 8.10 g ai/ha treatment at 7 days after application was (77.34 ± 5.50)%. Seed treatment with thiamethoxam also demonstrates highly effective control of thrips throughout the entire corn growing season [24]. This insecticide exhibits strong toxicity against *Frankliniella invasor* (Sakimura) (Thysanoptera: Thripidae), with an LC_50_ of 0.846 μg/mL after 24 h treatment [25]. This value is lower than the LC_50_ of thiamethoxam against *M. glycines* measured in this study. Thiamethoxam breaks down into clothianidin within the pest’s body [26]. Clothianidin exhibits potent agonistic activity at the Tp*α*2/r*β*2 site on nicotinic acetylcholine receptors (nAChRs) in *Thrips palmi* (Karny) (Thysanoptera: Thripidae), which may represent a key target site for the drug’s lethal effect on thrips [27]. The LC_50_ value of sulfoxaflor for *Megalurothrips usitatus* (Bagnall) (Thysanoptera: Thripidae), a major thrips pest of leguminous crops in Hainan Province, was 165.991 mg/L. Following exposure to the LC_25_ concentration, the CYP6EB24 gene within the cytochrome P450 family was upregulated by 9.6-fold [28]. In this study, the LC_50_ value of this pesticide against *M. glycines* was 15.72 mg/L, which is lower than the reported value. Previous study on *T. flavus* showed that acetamiprid, sulfoxaflor, and imidacloprid exhibited high control efficacy (more than 90%) in the pot trials after 7 days of application [9]. In this study, the control efficacy of the three insecticides at the highest concentration was (88.16 ± 8.83)%, (86.58 ± 4.50)%, and (90.52 ± 4.50)%, respectively.

Thrips from different regions exhibit varying sensitivities to insecticides. For example, the lowest LC_50_ value for imidacloprid against *F. intonsa* in cotton fields across different regions of Xinjiang Province was only 1.87 mg/L. In contrast, the toxicity of imidacloprid to *F. intonsa* in cotton fields across different regions showed significant variation, with LC_50_ values ranging from 4.36 to 143.93 mg/L [29]. Therefore, subsequent studies should further monitor the sensitivity of different geographical populations of *M. glycines* to commonly used pesticides. By integrating local pesticide application histories with resistance backgrounds, rational crop rotation strategies can be developed to provide a basis for regional integrated pest management.

Fenthion belongs to the class of organophosphorus compounds [30]. This exhibits good insecticidal efficacy against *T. flavus*, with an LC_50_ of only 2.26 mg/L, which is lower than the LC_50_ value for the *M. glycines* in this study [9]. Both abamectin and spinetoram belong to biological pesticides. Abamectin has a good insecticidal effect on *Thrips parvispinus* (Karny) (Thysanoptera: Thripidae), a pest species on ornamental plants in the United States [31]. Following treatment with sublethal concentrations of spinetoram, the MusABCH3 gene in the ATP-binding cassette (ABC) protein of *M. usitatus* was significantly upregulated by 9.33-fold [32]. The use of insecticides may also alter the competitive relationships among thrips species. For instance, after application of beta-cypermethrin, *Frankliniella occidentalis* (Pergande) (Thysanoptera: Thripidae) replaced *T. tabaci* as the predominant thrips species on cabbage [33]. Pyridaben is a widely used insecticide for controlling phytophagous mites, whiteflies, aphids, and thrips [34]. Its LC_50_ against *T. flavus* in soybean fields is 22.75 mg/L, which is similar to this study [9].

It is noteworthy that in the pesticide hazard classification recommended by the World Health Organization, imidacloprid, thiamethoxam, clothianidin, acetamiprid, sulfoxaflor, fenthion, and pyridaben are classified as moderately hazardous pesticides. Spinetoram is considered unlikely to cause acute hazards, while abamectin is classified as a highly hazardous pesticide [35]. Abamectin and Spinetoram, both botanical insecticides, demonstrated an efficacy exceeding 85% against *M. glycines* in pot trials seven days post-application. Despite not yet being registered for use in Chinese soybean fields, their potential as rotational treatments for controlling *M. glycines* warrants serious consideration. Although these pesticides are highly effective, their adverse effects on non-target organisms warrant systematic attention and evaluation. [34,36]. Given the current lack of registered pesticides for thrips in soybean fields, and the risk of pesticide resistance among farmers who rely on grain and vegetables for rotating pesticides [8], when the yellow sticky boards trapping method detects that the population of *M. glycines* reaches the economic threshold, preventive intervention should be taken during the initial peak period (about mid July, based on the 16% percentile division), rather than waiting for the thrips population peak during the peak flowering stage. Relying on sticky boards to capture and measure flight activity of thrips, rather than absolute population density or losses caused by feeding and spawning, future work should link these counts with plant damage assessment to establish robust economic thresholds. Although *M. glycines* is an emerging pest, much of its basic biology, such as its overwintering strategies and host range outside the soybean growing season, remains largely unknown. In addition, several unresolved issues remain to be addressed in future research, including: (1) the life history and biology of *M. glycines*; (2) the potential application of biological control technologies; (3) the detection of plant virus transmission by *M. glycines*; and (4) the monitoring and management of insecticide resistance.

## 5. Conclusions

This study systematically elucidated the field population dynamics of *M. glycines* and screened effective insecticides for its management. Results demonstrated that the pest exhibited a typical rise-and-fall pattern during the soybean growing season, with rapid population growth from flowering to pod-filling stages. The initial peak, peak occurrence, and late peak periods were concentrated in mid-July, late July to early August, and mid-to-late August, respectively, with yellow sticky traps proving more suitable for field monitoring. Laboratory bioassays, pot trials and field efficacy trials indicated that two neonicotinoid insecticides, thiamethoxam and clothianidin, exhibited excellent toxicity and field control efficacy, significantly outperforming conventional agents such as imidacloprid and abamectin. This study identifies the flowering stage as the primary critical control window, with the podding stage serving as a secondary alternative. These findings fill the current gap in registered control products for this pest in China and provide essential data to support scientific management strategies and future insecticide registration.

## Figures and Tables

**Table 1:** Concentration gradients for laboratory bioassay.

Insecticides	Concentration (mg·L^−1^)				
	I	II	III	IV	V
Thiamethoxam 30% SC	6.0	12.0	18.0	240	30.0
Clothianidin 48% SC	6.0	12.0	18.0	240	30.0
Sulfoxaflor 35% SC	10.0	15.0	20.0	25.0	30.0
Acetamiprid 25% EC	5.0	15.0	25.0	35.0	45.0
Imidacloprid 70% WP	10.0	20.0	30.0	40.0	50.0
Fenthion 50% EC	10.0	18.0	26.0	34.0	42.0
Pyridaben 30% EC	20.0	25.0	30.0	35.0	40.0
Abamectin 5% EC	20.0	25.0	30.0	35.0	40.0
Beta-cypermethrin 4.5% ME	30.0	50.0	60.0	70.0	80.0
Spinetoram 6% SC	50.0	60.0	70.0	80.0	90.0

**Table 2:** Concentration gradient for pot trials.

Insecticides	Concentration (g ai/ha)				
	I	II	III	IV	V
Thiamethoxam 30% SC	1.62	3.24	4.86	6.48	8.10
Clothianidin 48% SC	2.60	5.18	7.78	10.37	12.96
Sulfoxaflor 35% SC	1.98	2.97	3.96	4.95	5.94
Acetamiprid 25% EC	1.13	3.38	5.63	7.88	10.13
Imidacloprid 70% WP	6.30	12.60	18.90	25.20	31.50
Fenthion 50% EC	4.50	8.10	11.70	15.30	18.90
Pyridaben 30% EC	5.40	6.75	8.10	9.45	10.80
Abamectin 5% EC	0.90	1.13	1.35	1.58	1.8
Beta-cypermethrin 4.5% ME	1.22	2.03	2.43	2.84	3.24
Spinetoram 6% SC	2.70	3.24	3.78	4.32	4.86

**Table 3:** Occurrence periods of *Mycterothrips glycines* (Changchun, 2024–2025).

Year	Sticky Board Color	Initial Peak Period	Peak Occurrence Period	Late Peak Period	Mathematical Expressions	*R* ^2^	RMSE
2024	Yellow	11 July	29 July	10 August	$y=\frac{2616.8958}{1+e^{-0.101154\;\times (x-210.43)}}$	0.9972	52.7125
2024	Blue	23 July	5 August	14 August	$y=\frac{2032.9659}{1+e^{-0.119392\;\times (x-215.36)}}$	0.9957	51.5082
2025	Yellow	16 July	1 August	27 August	$y=\frac{5710.5002}{1+e^{-0.079742\;\times (x-212.01)}}$	0.9931	188.0151
2025	Blue	13 July	7 August	31 August	$y=\frac{3555.3406}{1+e^{-0.063941\;\times (x-218.65)}}$	0.9950	90.3051

Note: In the mathematical expressions, *x* represents the time dimension, describing the duration of the thrips population growth, and *y* represents the cumulative number of thrips individuals.

**Table 4:** Toxicity of ten insecticides against *Mycterothrips glycines* (Changchun, Jilin Province, 2024).

Insecticide	Probit Model	Correlation Coefficient	LC50 (mg/L) 95% Confidence Interval	Relative Bioactivity Index	Chi-Square
Thiamethoxam 30% SC	*y* = 3.4445*x* + 1.3068	0.98	12.87 11.78~13.89	4.73	5.22
Clothianidin 48% SC	y = 2.3474*x* + 2.3501	0.96	13.46 11.88~15.02	4.52	7.05
Sulfoxaflor 35% SC	y = 5.0477*x* − 1.0393	0.98	15.72 14.68~16.67	3.87	9.09
Acetamiprid 25% EC	y = 4.1324*x* + 0.0164	0.98	16.04 14.54~17.42	3.80	6.37
Imidacloprid 70% WP	y = 2.3074*x* + 1.9905	0.97	20.15 17.52~22.62	3.02	6.67
Fenthion 50% EC	y = 4.3363*x* − 0.3461	0.98	20.58 19.34~21.75	2.96	5.57
Pyridaben 30% EC	y = 6.9876*x* − 4.6695	0.98	24.20 22.84~25.34	2.52	6.96
Abamectin 5% EC	*y* = 5.6614*x* − 3.3092	0.99	29.36 28.01~30.79	2.07	2.58
Beta-cypermethrin 4.5% ME	*y* = 3.9825*x* − 1.7914	0.96	50.73 47.14~54.13	1.20	7.56
Spinetoram 6% SC	*y* = 8.7815*x* − 10.6750	0.98	60.88 58.41~63.02	1.00	7.47

**Table 5:** Insecticide efficacy of ten insecticides against *Mycterothrips glycines* in a pot trial (Changchun, Jilin Province, 2024).

Insecticides	Dose (g ai/ha)	Insecticide Efficacy (%)		
		Days After Application (day)		
		1	3	7
Thiamethoxam 30% SC	1.62	31.39 ± 10.45 b	46.75 ± 7.21 c	54.21 ± 10.29 c
	3.24	48.91 ± 8.16 ab	58.75 ± 3.75 bc	68.42 ± 5.58 bc
	4.86	55.47 ± 10.13 a	61.75 ± 10.41 b	73.16 ± 10.22 ab
	6.48	56.20 ± 10.64 a	70.75 ± 9.71 ab	83.42 ± 13.50 ab
	8.10	64.23 ± 11.07 a	78.25 ± 4.89 a	91.32 ± 7.06 a
Clothianidin 48% SC	2.59	25.55 ± 10.51 c	40.75 ± 11.98 c	54.21 ± 5.99 c
	5.18	39.42 ± 7.57 bc	49.00 ± 8.22 bc	57.37 ± 10.22 bc
	7.78	48.18 ± 11.07 ab	58.75 ± 7.02 b	64.47 ± 10.81 bc
	10.37	53.28 ± 9.79 ab	60.25 ± 8.63 b	72.37 ± 10.81 b
	12.96	64.23 ± 8.71 a	79.00 ± 8.22 a	92.10 ± 4.83 a
Sulfoxaflor 35% SC	1.98	13.87 ± 4.16 e	22.75 ± 4.28 d	37.63 ± 7.59 c
	2.97	24.82 ± 4.16 d	37.75 ± 4.28 c	60.53 ± 6.24 b
	3.96	37.23 ± 7.02 c	52.00 ± 7.21 b	74.74 ± 4.50 a
	4.95	54.01 ± 7.57 b	67.75 ± 4.28 a	79.47 ± 7.59 a
	5.94	67.88 ± 4.76 a	73.75 ± 8.39 a	88.16 ± 8.83 a
Acetamiprid 25% EC	1.125	10.95 ± 6.63 d	20.50 ± 6.16 d	43.95 ± 11.97 c
	3.375	25.55 ± 5.53 c	37.75 ± 7.78 c	61.32 ± 7.59 bc
	5.625	41.61 ± 9.30 b	59.50 ± 7.21 b	70.00 ± 11.37 ab
	7.875	54.01 ± 7.57 b	69.25 ± 6.16 b	82.63 ± 9.09 a
	10.125	75.18 ± 6.00 a	84.20 ± 6.16 a	86.58 ± 4.50 a
Imidacloprid 70% WP	6.3	29.93 ± 10.13 b	40.00 ± 11.56 c	51.84 ± 10.22 c
	12.6	40.88 ± 20.87 ab	52.00 ± 18.256 bc	66.05 ± 21.55 bc
	18.9	48.91 ± 12.90 ab	60.25 ± 8.63 abc	70.79 ± 5.99 abc
	25.2	56.20 ± 9.30 a	78.25 ± 13.88 a	77.89 ± 4.50 ab
	31.5	50.37 ± 7.57 ab	73.00 ± 8.13 ab	90.52 ± 4.50 a
Fenthion 50% EC	4.5	3.65 ± 4.90 d	13.75 ± 5.93 c	32.10 ± 9.42 c
	8.1	9.49 ± 7.02 cd	26.50 ± 8.63 c	37.63 ± 10.22 c
	11.7	20.44 ± 7.02 bc	42.25 ± 7.78 b	62.11 ± 11.71 b
	15.3	30.66 ± 8.16 ab	58.75 ± 8.39 a	71.58 ± 6.49 b
	18.9	42.34 ± 7.02 a	70.75 ± 7.21 a	90.53 ± 9.09 a
Pyridaben 30% EC	5.4	3.65 ± 5.53 d	17.50 ± 5.93 e	32.11 ± 7.59 c
	6.75	12.41 ± 5.77 cd	32.50 ± 5.93 d	50.26 ± 8.19 b
	8.1	23.36 ± 5.77 bc	47.50 ± 5.93 c	51.84 ± 7.59 b
	9.45	34.31 ± 5.77 b	62.50 ± 5.93 b	78.68 ± 10.67 a
	10.8	50.37 ± 8.40 a	77.50 ± 5.93 a	88.16 ± 6.24 a
Abamectin 5% EC	0.9	19.71 ± 9.30 a	31.00 ± 12.04 c	36.84 ± 10.06 c
	1.13	40.15 ± 11.99 b	49.00 ± 7.78 b	52.63 ± 9.26 bc
	1.35	47.45 ± 11.71 ab	52.00 ± 7.21 b	61.32 ± 9.00 b
	1.58	48.18 ± 3.05 ab	67.00 ± 4.89 a	84.21 ± 8.83 a
	1.8	58.39 ± 4.16 a	70.75 ± 5.56 a	89.74 ± 5.99 a
Beta-cypermethrin 4.5% ME	1.22	23.36 ± 7.74 d	41.50 ± 5.03 d	55.79 ± 9.42 b
	2.03	38.69 ± 4.76 c	54.25 ± 4.89 cd	61.32 ± 7.59 b
	2.43	48.91 ± 7.30 bc	58.75 ± 10.27 bc	73.16 ± 11.30 b
	2.84	54.01 ± 7.57 b	73.00 ± 14.86 ab	69.21 ± 10.22 ab
	3.24	70.80 ± 9.30 a	85.75 ± 5.56 a	89.74 ± 8.19 a
Spinetoram 6% SC	2.7	27.74 ± 6.53 b	37.00 ± 8.95 b	44.74 ± 9.26 c
	3.24	37.96 ± 9.30 b	45.25 ± 11.12 b	55.00 ± 12.67 bc
	3.78	45.26 ± 9.30 ab	49.00 ± 9.41 b	62.89 ± 10.29 bc
	4.32	48.91 ± 16.92 ab	57.25 ± 16.47 ab	72.37 ± 10.06 ab
	4.86	66.42 ± 13.75 a	74.50 ± 8.13 a	87.37 ± 5.85 a

Note: Values represent mean ± SD for each dose on each assessment day. One-way ANOVA was performed to assess differences among treatment groups. For each insecticide, different lowercase letters within a column denote significant differences between doses (Tukey’s test, *p* < 0.05). The same notation is used in the following tables.

**Table 6:** Field efficacy of imidacloprid, clothianidin and thiamethoxam against *Mycterothrips glycines* (Changchun, Jilin Province, 2023).

Insecticides	Dose (g ai/ha)	Field Efficacy (%)			
		Days After Application (day)			
		1	3	7	14
Thiamethoxam 30% SC	1.62	15.48 ± 3.44 b	40.47 ± 9.20 a	37.19 ± 6.83 b	43.23 ± 7.01 b
	3.24	66.13 ± 3.34 a	47.15 ± 5.84 a	75.47 ± 0.32 a	57.68 ± 9.33 ab
	4.86	61.14 ± 5.94 a	31.53 ± 20.43 a	70.35 ± 4.02 a	45.40 ± 17.61 b
	6.48	50.56 ± 8.97 a	64.78 ± 3.92 a	69.56 ± 3.46 a	63.11 ± 11.47 ab
	8.10	59.05 ± 11.17 a	67.73 ± 6.31 a	77.34 ± 5.50 a	85.89 ± 2.91 a
Clothianidin 48% SC	2.59	45.75 ± 7.77 ab	41.25 ± 4.83 a	41.59 ± 8.34 b	64.35 ± 6.43 a
	5.18	37.97 ± 5.85 ab	36.08 ± 8.28 a	56.52 ± 5.84 ab	79.50 ± 1.79 a
	7.78	30.31 ± 7.98 b	44.75 ± 13.11 a	74.26 ± 2.70 a	67.98 ± 4.90 a
	10.37	62.58 ± 13.41 ab	24.74 ± 6.08 a	67.44 ± 4.38 ab	79.52 ± 2.04 a
	12.96	69.20 ± 2.24 a	44.12 ± 5.15 a	68.83 ± 6.73 ab	80.94 ± 5.93 a
Imidacloprid 70% WP	6.3	56.46 ± 5.87 a	28.00 ± 1.89 a	34.40 ± 3.66 a	35.93 ± 4.31 a
	12.6	36.65 ± 5.49 a	30.29 ± 10.42 a	39.18 ± 2.44 a	37.27 ± 3.05 a
	18.9	47.73 ± 7.84 a	41.70 ± 9.91 a	45.50 ± 11.17 a	32.58 ± 8.14 a
	25.2	35.30 ± 8.23 a	52.34 ± 4.57 a	42.37 ± 0.92 a	38.11 ± 1.89 a
	31.5	39.04 ± 5.23 a	45.88 ± 5.56 a	51.22 ± 5.13 a	42.16 ± 0.70 a

Note: Values represent mean ± SE for each dose on each assessment day. One-way ANOVA was performed to assess differences among treatment groups. For each insecticide, different lowercase letters within a column denote significant differences between doses (Tukey’s test, *p* < 0.05).

## Data Availability

The original contributions presented in this study are included in the article/Supplementary Material. Further inquiries can be directed to the corresponding author.

## Supplementary Materials

The following supporting information can be downloaded at: https://www.mdpi.com/article/10.3390/insects17040365/s1, Table S1. Basic information of ten insecticides; Table S2. Meteorological Conditions from April to September 2024 to 2025; Table S3. Insecticide efficacy of ten insecticides after application at the highest concentration; Table S4. Regression model of *Mycterothrips glycines* population in relation to relative humidity and temperature; Figure S1. Schematic diagram of the laboratory bioassay; Figure S2. Logistic equation fitting of the daily cumulative population of *Mycterothrips glycines*; Figure S3. Regression analysis of *Mycterothrips glycines* population size in relation to relative humidity and temperature.

## Abbreviations

The following abbreviations are used in this manuscript:

*M. glycines*	*Mycterothrips glycines* Okamoto
*F. fusca*	*Frankliniella fusca* (Hinds)
*F. schultzei*	*Frankliniella schultzei* (Trybom)
*F. intonsa*	*Frankliniella intonsa* (Trybom)
*T. flavus*	*Thrips flavus* Schrank
*T. tabaci*	*Thrips tabaci* Lindeman
*M. usitatus*	*Megalurothrips usitatus* (Bagnall)
g ai/ha	g active ingredient/ha
