# Supplementary material for: Occurrence Dynamics and Chemical Control of Mycterothrips glycines in Soybean Field in Northeast China
Source: Insects. 2026 Mar 28;17(4):365. doi: 10.3390/insects17040365 (PMC13115614; doi:10.3390/insects17040365)
Supplement: Supplementary file 1 [file insects-17-00365-s001.zip › insects-4161765-supplementary.pdf]

Supplementary Materials

Article

Occurrence Dynamics and Chemical Control of *Mycterothrips glycines* in soybean field in Northeast China

Table S1. Basic information of ten insecticides.

Insecticide trade name	Common name	Formula tion	Group	WHO hazard classification	IRAC group	Mode of action	Commonly recommended dosage （g·hm^{-2}）	Manufacturer
Thiamethoxam 30% SC	thiamethoxam	30% SC	Neonicotino ids	Class II	4A	Nicotinic acetylcholine receptor (nAChR) competitive modulators	270	Hebei Zhongbao Green Crops Technology Co., Ltd., China
Clothianidin 48% SC	clothianidin	48% SC	Neonicotino ids	Class II	4A	Nicotinic acetylcholine receptor (nAChR) competitive modulators	375 (rice planthopper)	Qingdao Haina Bio-Tech Co.,Ltd., China
Sulfoxaflor 35% SC	sulfoxaflor	35% SC	Neonicotino ids	Class II	4C	Nicotinic acetylcholine receptor (nAChR) competitive modulators	300 (aphid)	Corteva Agriscience, China
Acetamiprid 25% EC	acetamiprid	25% EC	Neonicotino ids	Class II	4A	Nicotinic acetylcholine receptor (nAChR) competitive modulators	216	Hebei Runda Pesticide & Chemical Co.,Ltd., China

Imidacloprid 70% WP	imidacloprid	70% WP	Neonicotinoids	Class II	4A	Nicotinic acetylcholine receptor (nAChR) competitive modulators	90	Bayer CropScience China Co., Ltd., China
Fenthion 50% EC	Fenthion	50% EC	organophosphates	Class II	1B	Acetylcholinesterase (AChE) inhibitors	225	Pilarquim (Shanghai) Co., Ltd., China
Pyridaben 30% EC	pyridaben	30% EC	pyridazinone	Class II	21A	Mitochondrial complex I electron transport inhibitors	1 800	Dow Agrosciences China Ltd. (Hainan Branch)
Abamectin 5% EC	abamectin	5% EC	Abamectin	Class Ib	6	Glutamate-gated chloride channel (GluCl) allosteric modulators	82 (red spider)	Shanghai Yuelian Chemical Co., Ltd.
Beta-cypermethrin 4.5% ME	beta-cypermethrin	4.5% ME	Pyrethroids	—	3A	Sodium channel modulators	600 (*Artogeia rapae*)	Hebei Zhongbao Green Crops Technology Co., Ltd., China
Spinetoram $60 g\cdot L^{-1}$ SC	spinetoram	$60 g\cdot L^{-1}$ SC	Spinosyns	Class U	5	Nicotinic acetylcholine receptor (nAChR) allosteric modulators - Site I	900	Anhui Huaxing Chemical Industry Co., Ltd.

Table S2. Meteorological Conditions from April to September 2024 to 2025

Month	Average temperature (℃)		Precipitation (mm)		Relative humidity (%)	
	2024	2025	2024	2025	2024	2025
April	11.42	9.95	15.2	80.8	43.63	54.99
May	16.17	15.99	118.6	53.7	49.45	56.36
June	21.68	23.30	160.3	103.9	63.34	62.58
July	24.06	26.11	282.4	135.6	80.74	68.07
August	24.52	23.09	188.2	136.3	80.47	79.92
September	16.81	19.10	62.4	25.1	68.62	71.14

Table S3. Insecticide efficacy of ten insecticides after application at the highest concentration

Insecticides	Dose (g a.i.·hm^{-2})	Insecticide efficacy (%)		
		Days after application (day)		
		1	3	7
Thiamethoxam 30% SC	8.1	64.23±11.07ab	78.25±4.89ab	91.32±7.06a
Clothianidin 48% SC	12.96	64.23±8.71ab	79.00±8.22ab	92.10±4.83a
Sulfoxaflor 35% SC	5.94	67.88±4.76ab	73.75±8.39ab	88.16±8.83a
Acetamiprid 25% EC	10.125	75.18±6.00a	84.20±6.16ab	86.58±4.50a
Imidacloprid 70% WP	31.5	50.37±7.57bc	73.00±8.13ab	90.52±4.50a
Fenthion 50% EC	18.9	42.34±7.02c	70.75±7.21b	90.53±9.09a
Pyridaben 30% EC	10.8	50.37±8.40bc	77.50±5.93ab	88.16±6.24a
Abamectin 5% EC	1.8	58.39±4.16abc	70.75±5.56b	89.74±5.99a
Beta-cypermethrin 4.5% ME	3.24	70.80±9.30a	85.75±5.56a	89.74±8.19a
Spinetoram 60g·L^{-1} SC	4.86	66.42±13.75ab	74.50±8.13ab	87.37±5.85a

Table S4. Regression model of *Mycterothrips glycines* population in relation to relative humidity and temperature.

Year	Sticky board color	Meteorological factors	Regression model	R^2
2024	Yellow	Temperature	$y = 1.635213x^2 - 52.1443x + 403.24$	0.260
		Relative humidity	$y = 0.068644x^2 - 6.3366x + 156.17$	0.270
	Blue	Temperature	$y = 1.159843x^2 - 35.9643x + 268.40$	0.158
		Relative humidity	$y = 0.077247x^2 - 7.8184x + 199.78$	0.246
2025	Yellow	Temperature	$y = -0.509220x^2 + 30.0097x - 252.43$	0.017
		Relative humidity	$y = 0.063913x^2 - 1.9813 x + 0.18$	0.179
	Blue	Temperature	$y = -0.251357x^2 + 12.8111x - 60.47$	0.004
		Relative humidity	$y = 0.018829x^2 + 0.9871x - 55.26$	0.246

Note: *y* means thrips individuals per board.

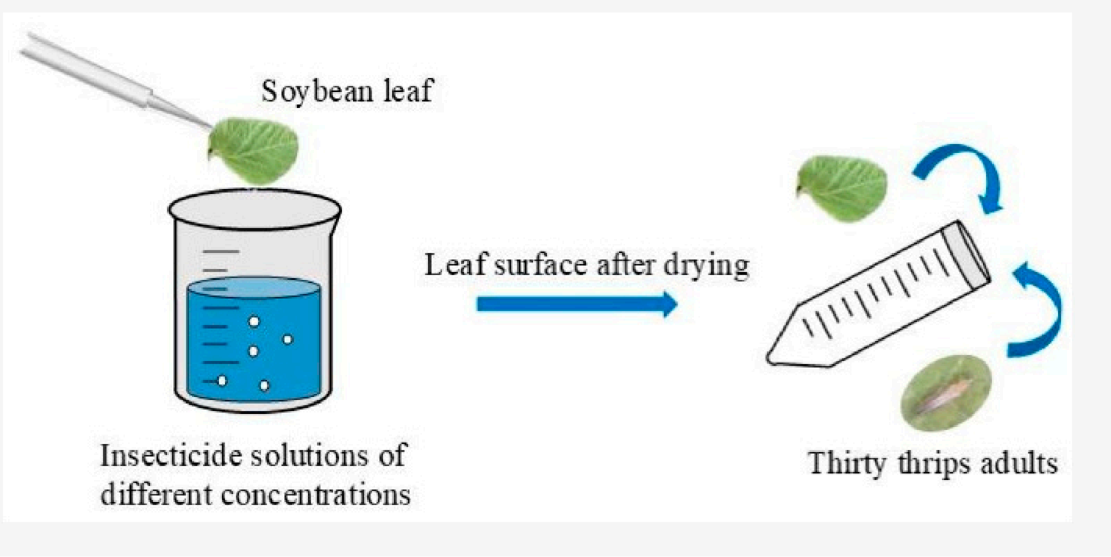

Figure S1. Schematic diagram of the laboratory bioassay.

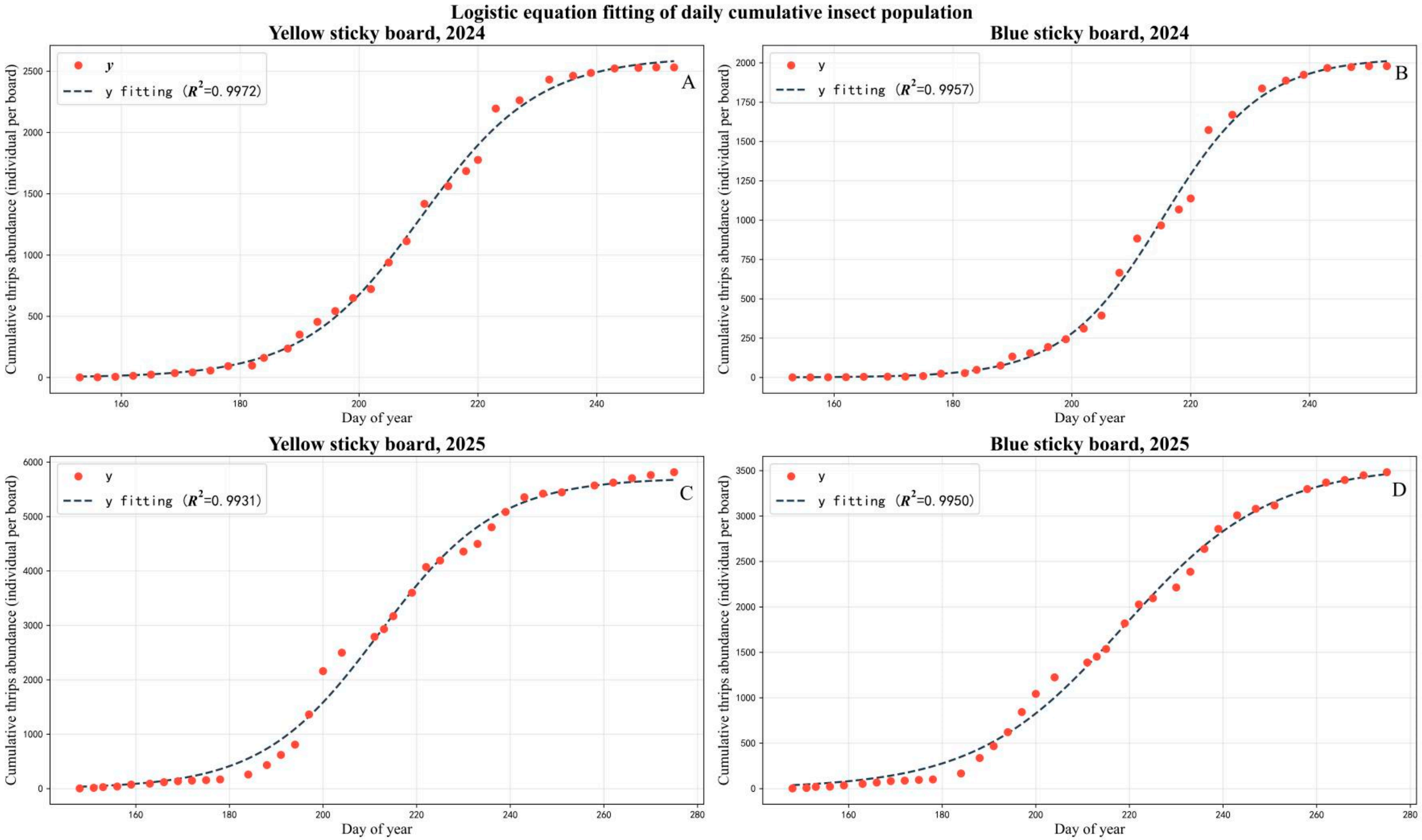

Figure S2. Logistic equation fitting of daily cumulative population of *Mycterothrips glycines*

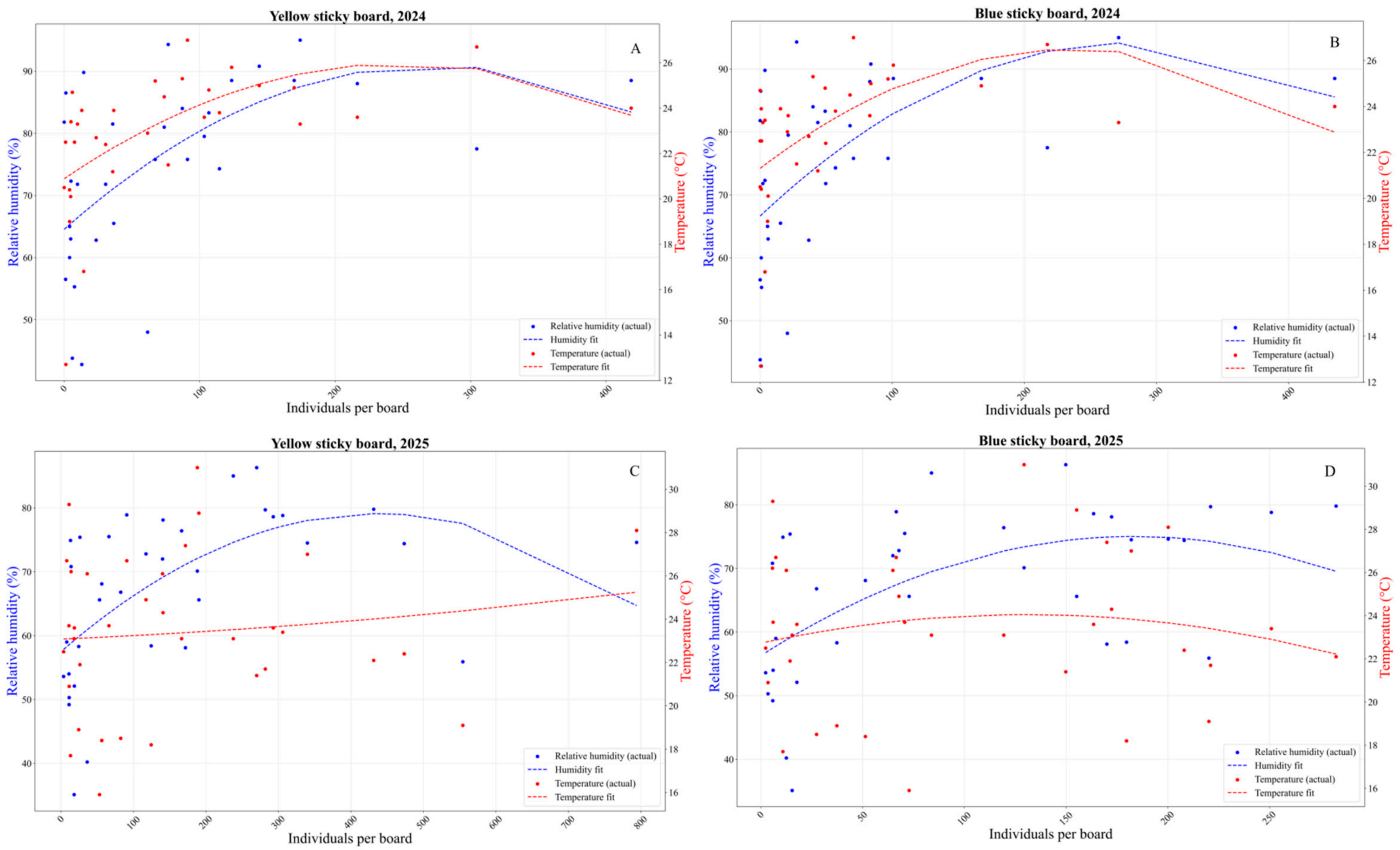

Figure S3. Regression analysis of *Mycterothrips glycines* population size in relation to relative humidity and temperature.
